# Pneumonia, influenza, and dengue cases decreased after the COVID-19 pandemic in Thailand

**DOI:** 10.1186/s41182-022-00419-2

**Published:** 2022-03-25

**Authors:** Rapeepun Prasertbun, Hirotake Mori, Aongart Mahittikorn, Sukhontha Siri, Toshio Naito

**Affiliations:** 1 Department of General Medicine, Juntendo University Faculty of Medicine, Tokyo, 113-8431 Japan; 2 Department of Protozoology, Faculty of Tropical Medicine, Mahidol University, 420/6 Ratchawithi Road, Ratchathewi, Bangkok, 10400 Thailand; 3 Department of Epidemiology, Faculty of Public Health, Mahidol University, 420/1 Rachawithi Road, Ratchathewi, Bangkok, 10400 Thailand

**Keywords:** COVID-19, Pneumonia, Influenza, Dengue, Thailand, Asia

## Abstract

The coronavirus disease 2019 (COVID-19) pandemic has affected all healthcare systems worldwide. Effective COVID-19 preventive measures, including wearing a mask, hand washing, avoiding the “Three Cs”, and city lockdowns, could decrease other infectious diseases. The case numbers of the major infectious diseases in Thailand were investigated (pneumonia, influenza, and dengue fever) during the COVID-19 pandemic using Thailand government national data sources from 2018 to August 2021. Pneumonia, influenza, and dengue fever cases decreased after the COVID-19 pandemic. In addition to respiratory tract infections, COVID-19 preventive measures could decrease dengue fever cases.

**Dear Editor**,

Coronavirus disease 2019 (COVID-19) is a global threat, and various social preventive measures have been taken in each country. Effective COVID-19 preventive measures, which are wearing a mask, washing hands, or alcohol hand hygiene, avoiding the “Three Cs” (closed spaces with poor ventilation, crowded spaces with many people nearby, and close-contact settings such as close-range conversations), and during a crisis, city lockdowns, could decrease the other infectious diseases [1, 2]. This study aimed to evaluate case numbers of the major infectious diseases in Thailand (pneumonia, influenza, and dengue fever) during the COVID-19 pandemic.

This retrospective cohort study used Thailand government national data sources from 2018 to August 2021. The annual case numbers of COVID-19, pneumonia, influenza, and dengue fever were examined using the database of the Ministry of Public Health in Thailand [3–5].

The number of COVID-19 cases from January 12, 2020, to December 3, 2021 was reported by the Ministry of Public Health; 2,130,641, and 20,880 people were infected and died, respectively. The COVID-19 cases did not increase from January 2020 to November 2020; the number of cases/day was less than 2,000. However, the cases increased rapidly in December 2020. The outbreak peaked in August 2021, with 589,415 cases, as shown in Figs. 1, 2 and 3. Restrictions on movement of people within the country were introduced from 26 March 2020 to 30 April 2020.

**Fig. 1:**
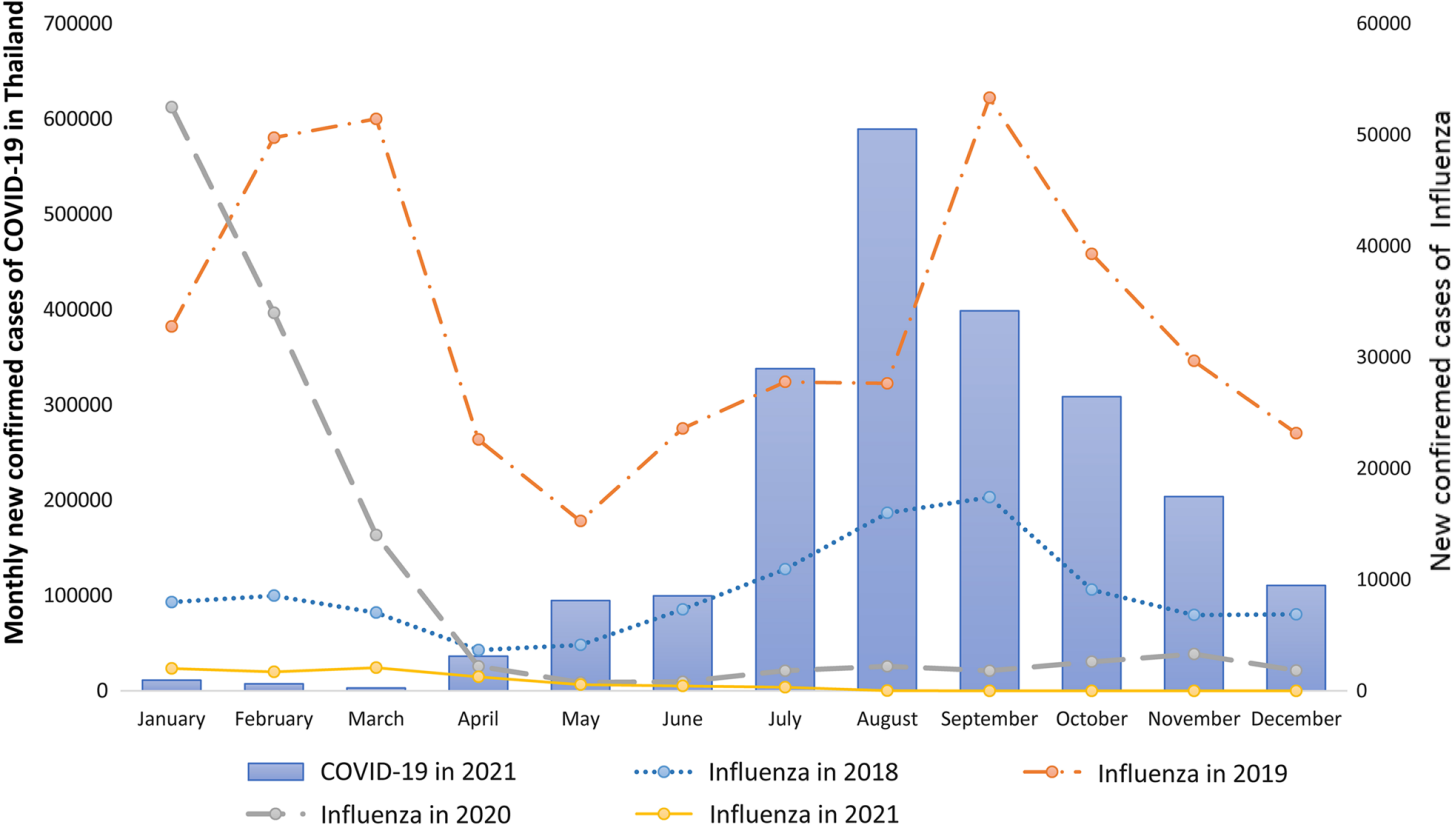
Influenza cases from 2018 to 2021 in Thailand

**Fig. 2:**
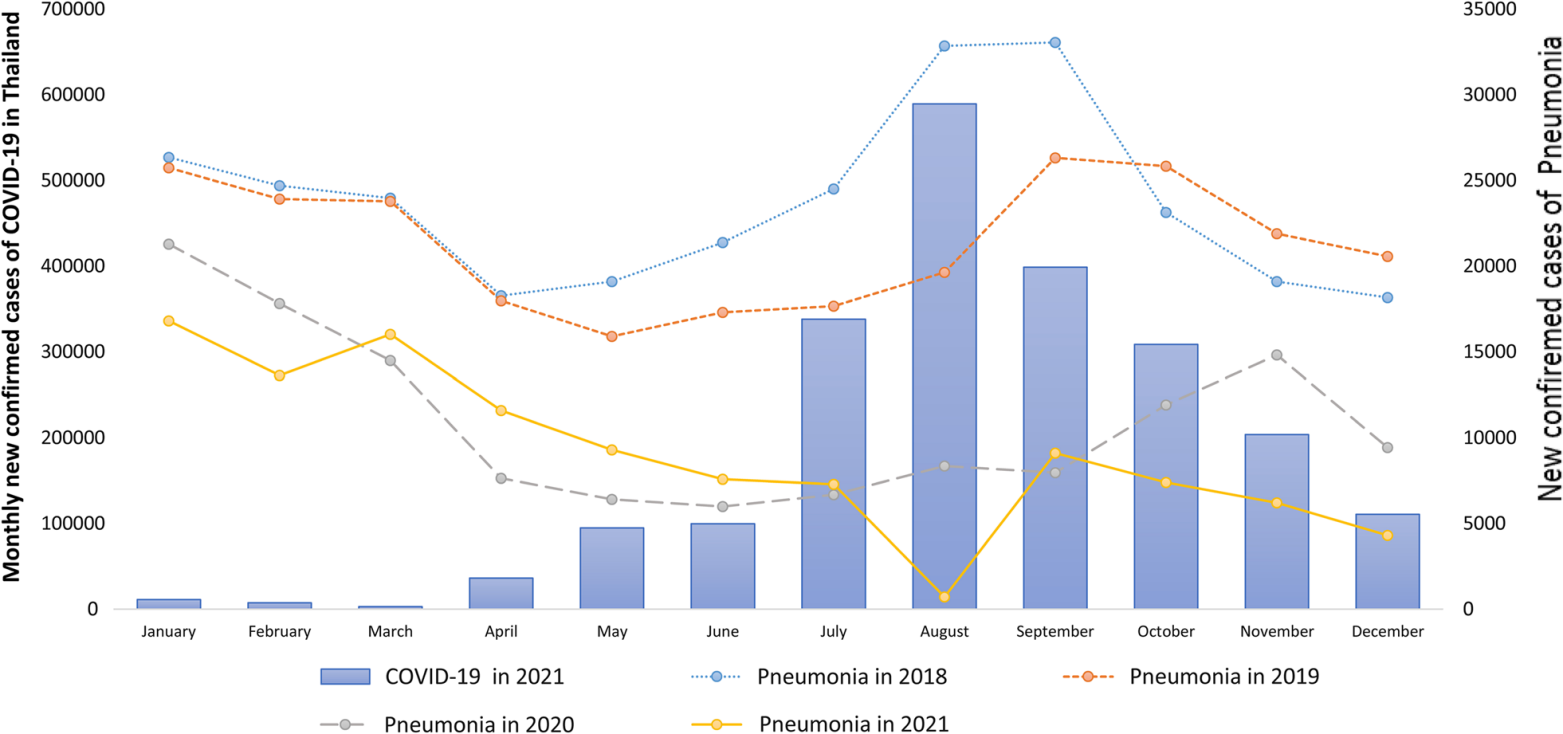
Pneumonia cases from 2018 to 2021 in Thailand

**Fig. 3:**
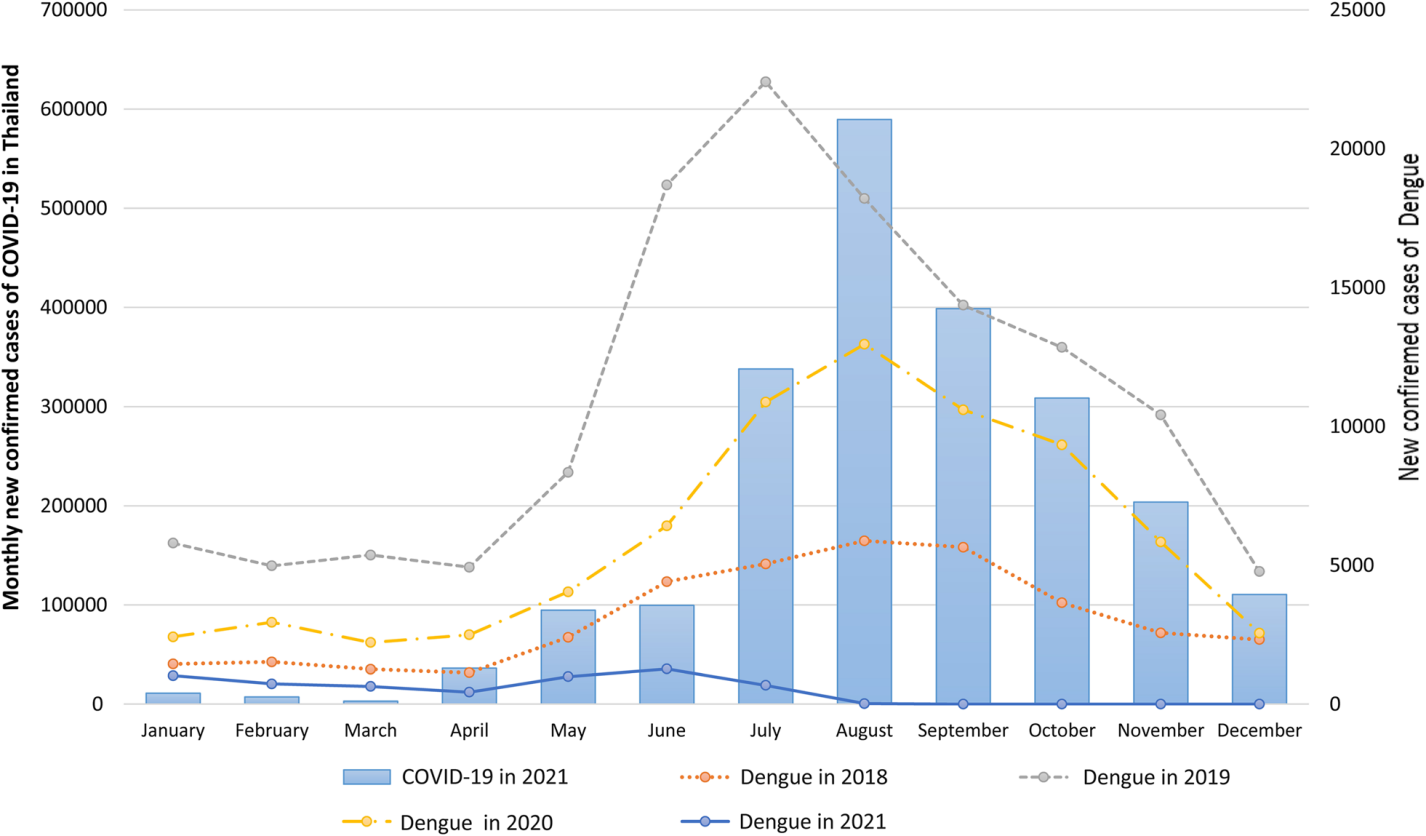
Dengue cases from 2018 to 2021 in Thailand

Influenza is endemic in Thailand year round, high in the rainy season from June to October and the winter season from January to March. In 2019, the maximum and minimum numbers of cases of influenza were in September (53,339) and May (15,275), respectively. However, after COVID-19 started in 2020, the incidence of influenza dropped rapidly from February to April. The incidence of influenza then remained lower, at less than 3,000 cases/month as shown in Fig. 1. [4]. The seasonal trend of pneumonia in the pre-COVID-19 era resembles that of influenza: high in the rainy season from June to October and in the winter season from January to March, as shown in Fig. 2. However, few pneumonia cases were reported from January to March in 2021, while COVID-19 was an epidemic. Dengue is highly prevalent in the summer and rainy season from May to October and decreases in winter from December to March, as shown in Fig. 3. In 2020, the number of dengue cases was lower than in 2019, and in 2021, fewer dengue cases were reported; less than 2000 cases were reported from May.

The decreased incidence of major respiratory infectious diseases worldwide has been reported elsewhere [6]. COVID-19 preventive measures, such as the changes in lifestyle, social lockdowns, and wearing facial masks, and immigration restrictions, are effective for other respiratory infections, such as pneumonia and influenza. The United States reported a 61% decrease in the number of specimens submitted and a 98% decrease in influenza activity as measured by the percentage of submitted specimens testing positive. In Japan, we previously reported a 44–53% reduction in community-acquired pneumonia admissions from April through September of 2020 [6]. During the COVID-19 pandemic, in addition to preventive measures against respiratory infections, all suspected COVID-19 and regular pneumonia cases were quickly sent to the hospital, diagnosed, and treated; the risk of infection with pneumonia was minimized.

Dengue cases decreased in Thailand in 2020 and 2021. Particularly in 2021, few dengue cases were reported. Regarding mosquito-borne diseases, malaria cases have reportedly increased, especially in African countries. However, dengue cases may decrease or increase during a COVID-19 pandemic. Dengue cases decreased significantly in Sri Lanka and China. COVID-19 lockdowns decreased dengue transmission in Sri Lanka [7]. In Singapore and India, however, the social distancing policy increased dengue cases. In Singapore, the increased time spent at home might increase exposure to Aedes mosquitos [8]. In India, the density of the immature Aedes mosquito increased drastically during the COVID-19 lockdown due to insufficient vector control programs [9]. In Thailand, the public health staff and the military were heavily involved with COVID-19 mitigation activities with less emphasis on dengue source reduction. However, the community engagement to actively remove breeding habitats in and around homes may have improved during extended periods spent at home during the lockdown. People had more time to pay attention to the vector breeding in their premises. Other possible factors are restrictions on the movement of people within the country, especially closed schools, universities, and offices to control COVID-19 transmission. Dengue infections were clustered among schools in Thailand [10]. Restrictions on the movement of people can result in reduced access to the mosquitoes.

The findings in this report are subject to limitations, including the lack of age-specific weekly data, which cannot distinguish between community and hospital patients, and other factors, such as the sharp reductions in global travelers and increased vaccine use, which might have played a role in decreasing disease spread.

In conclusion, pneumonia, influenza, and dengue fever cases decreased during the COVID-19 pandemic in Thailand. Vector-borne diseases, such as dengue fever could decrease, in addition to respiratory tract infections, due to preventive measures enacted during global pandemics.

## Data Availability

All the data used in this in this letter are drawn from the references provided.

## Abbreviation

COVID-19: Coronavirus disease 2019

## References

[1] Brueggemann AB, van Rensburg MJJ, Shaw D, McCarthy ND, Jolley KA, Maiden MC (2021). Changes in the incidence of invasive disease due to Streptococcus pneumoniae, Haemophilus influenzae, and Neisseria meningitidis during the COVID-19 pandemic in 26 countries and territories in the Invasive Respiratory Infection Surveillance Initiative: a prospective analysis of surveillance data. Lancet Digital Health.

[2] Uyeki TM, Wentworth DE, Jernigan DB (2021). Influenza activity in the US during the 2020–2021 season. JAMA.

[3] Ministry of Public Health, pneumonia situation in Thailand 2021 [Accessed 3 Dec 2021]. Available from: http://203.157.41.226/disease/Pneumonia.php.

[4] Ministry of Public Health, influenza situation in Thailand 2021 [Accessed 3 Dec 2021]. Available from: http://203.157.41.226/disease/InfluenzaFlu.php.

[5] Ministry of Public Health, dengue fever situation in Thailand 2021 [Accessed 3 Dec 2021]. Available from: http://203.157.41.226/disease/Denguefever.php.

[6] Yan Y, Tomooka K, Naito T, Tanigawa T (2022). Decreased number of inpatients with community-acquired pneumonia during the COVID-19 pandemic: a large multicenter study in Japan. J Infect Chemother.

[7] Liyanage P, Rocklöv J, Tissera HA (2021). The impact of COVID–19 lockdown on dengue transmission in Sri Lanka; a natural experiment for understanding the influence of human mobility. PLoS Negl Trop Dis.

[8] Lim JT, Chew LZX, Choo ELW, Dickens BSL, Ong J, Aik J (2021). Increased dengue transmissions in Singapore attributable to SARS-CoV-2 social distancing measures. J Infect Dis.

[9] Reegan AD, Gandhi MR, Asharaja AC, Devi C, Shanthakumar SP (2020). COVID-19 lockdown: impact assessment on Aedes larval indices, breeding habitats, effects on vector control programme and prevention of dengue outbreaks. Heliyon.

[10] Olanratmanee P, Wilder-Smith A, Byass P, Tozan Y, Dambach P, Quiñonez CA, Louis VR (2016). Spatial variations in Dengue transmission in schools in Thailand. PLoS ONE.

